# A novel variant in *SLC16A2* associated with typical Allan-Herndon-Dudley syndrome: a case report

**DOI:** 10.1186/s12887-022-03259-5

**Published:** 2022-04-05

**Authors:** Xiaodan Chen, Li Liu, Chunhua Zeng

**Affiliations:** Department of Genetics and Endocrinology, Guangzhou Women and Children’s Medical Center, Guangzhou Medical University, 9 Jinsui Rd, 510623 Guangzhou, China

**Keywords:** *SLC16A2*, Splicing variant, Allan-Herndon-Dudley syndrome, Thyroid hormone

## Abstract

**Background:**

Allan-Herndon-Dudley syndrome (AHDS) is an X-linked recessive neurodegenerative disorder caused by mutations in the *SLC16A2* gene that encodes thyroid hormone transporter. AHDS has been rarely reported in China.

**Case presentation:**

This study reported a novel splicing mutation in the *SLC16A2* gene in an 18-month-old male patient with AHDS. The patient was born to non-consanguineous, healthy parents of Chinese origin. He passed new-born screening for hypothyroidism, but failed to reach developmental milestones. He presented with hypotonia, severe mental retardation, dysarthria and ataxia. Genetic analysis identified a novel splicing mutation, NM_006517.4: c.431-2 A > G, in the *SLC16A2* gene inherited from his mother. The patient received Triac treatment, (triiodothyroacetic acid), a thyroid hormone analogue for 3 months. Triac treatment effectively reduced serum TSH concentrations and normalized serum T3 concentrations in the patient.

**Conclusions:**

This study reported the first case of AHDS treated by Triac in China. And the study expanded the mutational spectrum of the *SLC16A2* gene in AHDS patients.

**Supplementary information:**

The online version contains supplementary material available at 10.1186/s12887-022-03259-5.

## Background

Allan-Herndon-Dudley syndrome (AHDS) is an X-linked neurodevelopmental disorder, first described at 1944 by Allan et al. [1]. This disorder was caused by the deficiency of monocarboxylate transporter 8 (MCT8), encoded by the *SLC16A2* gene (Solute Carrier Family 16 Member 2) [2–4]. The *SLC16A2* gene is located in Xq13.2, formerly called monocarboxylate transporters 8 (*MCT8*) [5]. As one of the thyroid hormone transporters, MCT8 expresses ubiquitously and facilitate the uptake of thyroid hormone into cells. Particularly, MCT8 mediates transport of active T3 and T4 across blood-brain-barrier and into central neurons [6]. Through binding to its nuclear receptors, thyroid hormone plays an important role in brain development and function. MCT8 deficiency due to *SLC16A2* variant causes AHDS characterized by mental and motor developmental delay, and thyroid functional abnormalities of high serum T3, reduced T4, and normal or mildly increased Thyroid stimulating hormone (TSH) [7]. To date, a total of 159 variants have been reported, mostly missense/nonsense and insertion/deletion variants. Only few variants in splicing sites in the *SLC16A2* gene were reported [5, 8–12]. The aim of the current study is to report a novel splicing variant in the *SLC16A2* gene in a Chinese patient with AHDS and the hormonal effects of Triac in this patient.

## Case presentation

The patient, an eighteen-month-old Chinese boy, was the first child born to healthy parents after uneventful pregnancy and delivery. His birth weight was 3 kg, birth height was 50 cm, and birth head circumstance was 34 cm. He passed hearing test and newborn screening for hypothyroidism after birth. He experienced feeding difficulty since his birth. At 6-month-old he presented with global hypotonia and developmental delay. He had never been able to hold up his head and could not sit. Physical examination revealed hypotonia in the trunk and hypertonia in the extremities. He had elongated face with bifrontal narrowing and flat nose, otherwise didn’t show dysplastic ears, pectus excavatum, scoliosis, flat feet, or lateral deviation of great toe etc. Thyroid function test in local medical center was performed as part of routine evaluation of global developmental delay, demonstrating increased free triiodothyronine (FT3) of 9.49 pg/ml (normal range 2.00-4.40 pg/ml), decreased free thyroxine (FT4) of 0.49 pg/dl (normal range 1.00-1.70 pg/dl) and mildly increased thyroid-stimulating hormone concentration (TSH) of 4.93 µIU/ml (normal range 0.27–4.20 µIU/ml) (Table 1). Similar symptoms were not observed in other family members. Further ultrasonography showed a normal thyroid gland. Brain magnetic resonance imaging (MRI) showed delayed myelination and mild cerebral atrophy. He was diagnosed as hypothyroidism and treated with levothyroxine sodium for 2 months. The patient discontinued levothyroxine sodium due to no improvement.

**Table 1:** Clinical features of the AHDS patient compared with a patient with different splicing mutation

Patients	The case in the current study					The case in the literature [8]
	Before Triac treatment			After Triac treatment		
Age	6-month-old	18-month-old			24-month-old	8-month-old
Mutation	c.431-2 A > G					c.431-1G > C
Ethnicity	Chinese					Chinese
Gender	Male					Male
Age of diagnosis	12-month-old					8-month-old
Family history	No					Yes
Hypotonia	Yes		Yes	Yes		Yes
Dystonia	Yes		Yes	Yes		Yes
Head control	No		Improved	Improved		No
Ability to sit	No		No	No		No
Language development	No		No	No		No
Cognitive dysfunction	Yes		Mildly improved	improved		Yes
Hearing impairment	No		No	No		Not available
Seizure	No		No	No		Not available
Serum FT3	9.49 pg/ml		8.12 pg/ml	7.35pg/ml		7.12 pg/ml
(normal range)	(2.00-4.40)		(2.66–4.82)	(4.10–7.42)		(2.41–5.50)
Serum FT4	0.49 pg/dl		0.68 pg/dl	0.07 pg/dl		0.87 pg/dl
(normal range)	(1.00-1.70)		(1.12–1.77)	(0.19–0.29)		(0.96–1.77)
Serum TSH	4.93 uIU/ml		5.33 uIU/ml	0.854 uIU/ml		5.55 uIU/ml
(normal range)	(0.27–4.20)		(0.38–7.31)	(0.38–7.31)		(0.70–5.97)
Brain MRI	delayed myelination and mild cortical atrophy		delayed myelination and mild cortical atrophy	NA		delayed myelination and severe cortical atrophy

When the patient was 12-month-old, he was not able to hold up his head, and could not sit and stand. He presented with severe global developmental delay, and generally could not reach any milestones. Physical examination revealed hypotonia in the trunk and hypertonia in the extremities. He had elongated face with bifrontal narrowing and flat nose. His height (75.4 cm) just reached − 2 SD (73 cm) and his weight (7 kg) was below − 2 SD (8.2 kg) on growth chart. The head circumference was 45 cm (-2 SD 44.2 cm) and chest circumference was 44 cm. Thyroid function test revealed increased FT_3_ of 8.86 pg/ml (normal range 2.66–4.82 pg/ml), decreased FT_4_ of 0.94 pg/dl (normal range 1.12–1.77 pg/dl), and normal TSH of 0.47 µIU/ml (normal range 0.38–7.31 µIU/ml). Euthyroxol was prescribed with methimazole to the patient for 3 months and then stopped due to no effect.

**Fig. 1:**
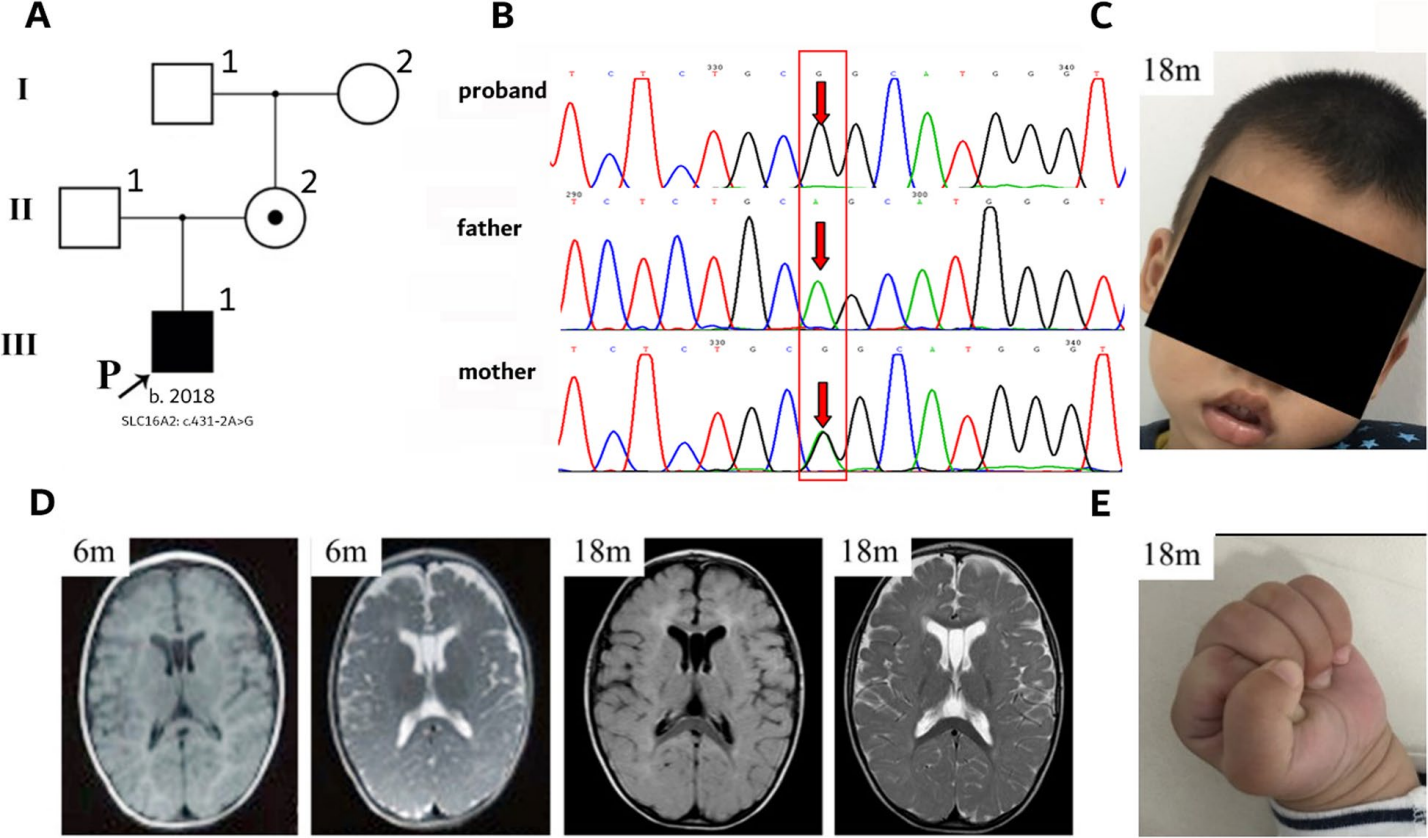
Characteristics of the 18-month-old male patient with AHDS. **A** Pedigrees of the AHDS patient. He harbored the variant from his healthy mother. Symbols and nomenclature follow standardized human pedigree nomenclature [13]. **B** Chromatograms of *SLC16A2* variants identified in the patient. **C** Photograph of the patient at 18-month-old. He couldn’t hold his head steady. **D** MRI of the patient showed delayed myelination and mild cortical atrophy at 6-months old and worsening cortical atrophy at 18-months old. E, Dystonic posturing of the hands

Direct sequencing of the *SLC16A2* gene was applied to coding sequences of all exons of the *SLC16A2* gene (NM_006517.4.) with exon/intron boundaries when the patient was 12-month-old. DNA was extracted using QIAamp DNA Blood Mini Kit (250) (QIAGEN, German). DNA as a template was amplified by PCR with primers designed by Primer 5.0 software program (Table S1). Sanger sequencing was performed in *SLC16A2* gene. PCR amplified and sequenced using an ABI 3500 amplify instrument and a Genetic Analyzer 3130 (Applied Biosystems). Genetic analysis revealed the presence of a novel splicing variant (NM_006517.4: c.431-2 A > G). By using online programs FATHMM, NetGene2, Human Splicing Finder 3.1 and Mutation Taster, the pathogenicity of the variant c.431-2 A > G was predicted to be deleterious by causing splice acceptor site change. The variant c.431-2 A > G may result in splicing change at protein level. Pedigree analysis revealed that this variant was inherited from his heterozygous healthy mother, but was not found in his maternal grandparents (Fig. 1A/B). His heterozygous mother had normal thyroid function.

At 18-month-old, the patient showed mild motor improvement. He had better head control and better eye to eye contact communication however, he was still not able to sit, walk and speak. He had mild feeding difficulty and drooling. He presented with hypotonia in the trunk and hypertonia in the extremities with clenched fists (Fig. 1C/E). Thyroid function test showed increased FT_3_ of 8.12 pg/ml (normal range 2.66–4.82 pg/ml), decreased FT_4_ of 0.68 pg/dl (normal range 1.12–1.77 pg/dl), and normal TSH of 5.33 µIU/ml (normal range 0.38–7.31 µIU/ml) (Table 1). Brain MRI showed thin corpus callosum, delayed myelination and cerebral atrophy (Fig. 1D).

Triac, a T3 analog, has been reported as a promising candidate to normalize serum T3 levels and thus alleviate the thyrotoxicosis in the patient with MCT8 deficiency. With a written and signed informed consent, Triac treatment has been initiated to treat the patient and maintained for 3 months. Triac dose started with 10 µg/kg (0.0875 mg) per day, doubling the dose (0.17 mg per day) after 2 weeks. After Triac treatment with the dose of 0.17 mg per day for 3 months, his height (82.4 cm) and weight (8.7 kg) were still mildly below − 2 SD (83 cm and 9.6 kg) on growth chart. The head circumference (48 cm) was in normal range and chest circumference reached 46.5 cm. Thyroid function test showed normal FT3 of 7.35 pmol/L (normal range 4.10–7.42 pmol/L), decreased FT_4_ of 5.19 pmol/L (normal range 14.45–22.74 pmol/L) and normal TSH of 0.854 µIU/ml (normal range 0.38–7.31 µIU/ml). And no abnormality was found in EEG (Electroencephalogram) and B-ultrasound of abdominal organs.

According to Human Gene Mutation Database (HGMD), a total of 5 splicing variants in the *SLC16A2* gene have been reported, one of which carried a variant in the same intron with the case in this study [8]. To further characterize the novel splicing variant in this case, we compared the clinical and mutational features between these two cases (Table 1). Both cases presented with typical phenotype of AHDS.

## Discussion and conclusions

We describe a Chinese patient diagnosed with AHDS, carrying a new splicing variant in the *SLC16A2* gene never reported so far. The patient exhibited severe development delay, thyroid function abnormalities of elevated FT3 and decreased FT4, and delayed brain myelination, indicating the novel splicing variant c.431-2 A > G in the *SLC16A2* gene is associated with severe phenotype of AHDS. To date, of 159 variants in the *SLC16A2* gene, the splicing variants were reported in only few AHDS cases [5, 8, 9, 12]. Through literature review, one case with a splicing variant located close to c.431-2 A > G in the *SLC16A2* gene has been reported [8]. An 8-month-old Chinese boy with AHDS carrying a splicing variant c.431-1G > C in the *SLC16A2* gene presented with severe intellectual and motor developmental delay, delayed myelination of the white matter and elevated serum FT3 level. These results indicate the splicing site of c.430–431 in the *SLC16A2* gene may be the key point for splice acceptor site. Further functional study is needed.

The T3 analog Triac has been reported as a promising candidate to normalize serum T3 levels and thus alleviate the thyrotoxicosis, and restore thyroid hormone signaling in the brain [3, 14]. There were no reports about the patients with MCT8 deficiency treated with Triac in China. Therefore, we refer to the literature published in 2014 by Dumitrescu, AM, et al., Triac therapy began at 0.0875 mg per day [3]. And we doubled the dose after 2 weeks. After 3 months of Triac treatment, the patient’s FT_3_ and TSH were back to normal.

Because of reduced FT4 and mildly increased TSH levels, some patients with AHDS have been initially suspected as hypothyroidism and treated with levothyroxine, just like the patient in the current study [15]. Although no beneficial effect on mental and motor function observed with levothyroxine, some improvements have been observed in body weight and heart rate due to amelioration of peripheral hyperthyroidism [15]. No significant improvement of mental and motor function were observed in our patient after 3-months Triac treatment.

The study supports that the novel splice acceptor site variation c.431-2 A > G in *SLC16A2* gene is pathogenic, which is associated with typical phenotype of AHDS. Triac treatment effectively reduced serum TSH concentrations and normalized serum T3 concentrations in the patient. This is the first case of MCT8 deficiency treated by Triac in China.

## Supplementary Information

**Additional file 1.**

## Data Availability

The variant data were deposited in ClinVar (https://www.ncbi.nlm.nih.gov/clinvar/ Submission ID: SUB11199328, NCBI tracking system #16,854,978, an SCV accession SCV002106318). Other data that support the findings of this study are available from the corresponding author upon reasonable request.

## Abbreviations

AHDS: Allan-Herndon-Dudley syndrome
MCT8: Monocarboxylate transporter 8
MRI: Magnetic resonance imaging
T3: Triiodothyronine
T4: Thyroxine
TSH: Thyroid stimulating hormone concentration
MRI: magnetic resonance imaging
HGMD: Human Gene Mutation Database
