# Supplementary material for: A novel variant in SLC16A2 associated with typical Allan-Herndon-Dudley syndrome: a case report
Source: BMC Pediatr. 2022 Apr 5;22:180. doi: 10.1186/s12887-022-03259-5 (PMC8981932; doi:10.1186/s12887-022-03259-5)
Supplement: Supplementary file 1 — Additional file 1. [file 12887_2022_3259_MOESM1_ESM.docx]

Table S1 The primers and PCR conditions for *SLC16A2* gene

| **Exon** | **Primer** | **Sequence** | **Annealing temp[°C]** | **Length of product** |
| --- | --- | --- | --- | --- |
| 1 | 1F  1R | CTGTAGCGGCTGCCTGTTGA  ACGCCTTGCACCTAGTCTCC | 60 | 942 |
| 2 | 2F  2R | GACAAGCCTGACCTTAGCTC  CCCAGCTCTACGAACTACCAG | 57 | 741 |
| 3 | 3F  3R | AGTCCAAGTTTTGCTGATTCCC  GCCCTTTCAATGTCTCCCAC | 60 | 795 |
| 4 | 4F  4R | CAGGAAAGTAAGCAGTAGGGG  AAAGCAGGTGAAATGGAGGC | 57 | 389 |
| 5 | 5F  5R | CTAACCATTCAGCCCAACTACTC  GAACTGTCAGAAAGCCAAACTCA | 59 | 691 |
| 6 | 6F  6R | CTGGATAGGCACTGTGATGG  GAGTTTGGAACACCTGGAGC | 55 | 482 |

| PCR conditions | | | |
| --- | --- | --- | --- |
| Step | Temperature  [°C] | Times | Cycles |
| 1 | 95 | 5min | 1 |
| 2 | 95 | 30sec | 36 |
| 3 | Annealing temp | 30sec |  |
| 4 | 72 | 50sec |  |
| 5 | 72 | 10min | 1 |
